# Ketogenic Diet Exacerbates L-Arginine-Induced Acute Pancreatitis and Reveals the Therapeutic Potential of Butyrate

**DOI:** 10.3390/nu15204427

**Published:** 2023-10-18

**Authors:** He Xia, Jing Guo, Jian Shen, Shiman Jiang, Shengyi Han, Lanjuan Li

**Affiliations:** State Key Laboratory for Diagnosis and Treatment of Infectious Diseases, National Clinical Research Center for Infectious Diseases, National Medical Center for Infectious Diseases, Collaborative Innovation Center for Diagnosis and Treatment of Infectious Diseases, The First Affiliated Hospital, Zhejiang University School of Medicine, Hangzhou 310003, China; xiahe@zju.edu.cn (H.X.); guojingzju@163.com (J.G.); shiman_j@zju.edu.cn (S.J.); 21918241@zju.edu.cn (S.H.)

**Keywords:** acute pancreatitis, ketogenic diet, L-arginine, butyrate

## Abstract

The ketogenic diet (KD) has emerged as a popular weight-loss regimen in recent years. However, it has been confirmed to elicit a mild inflammatory response in the intestinal epithelium and exacerbate various digestive disorders. The severity of acute pancreatitis (AP) is closely associated with the permeability of the intestinal epithelium and gut microbiota, yet the impact of KD on acute pancreatitis remains unclear. In this study, we induced acute pancreatitis using L-arginine in mice fed with KD. The consumption of KD resulted in an elevation of lipopolysaccharide-binding protein (LBP), accompanied by upregulated cytokines (IL-1a, IL-5, IL-12, MIP-1a, and Rantes) and dysfunction of the intestinal barrier both in control and AP groups. The bloom of *Lachnospirales* and *Erysipelotrichales* was observed as a specific profile of gut microbiota in KD-fed mice with AP, along with downregulation of carbohydrate metabolism and depletion of short-chain fatty acids (SCFAs). Antibiotic decontamination reduced the cytokine storm and tissue necrosis but did not significantly improve the integrity of the intestinal barrier in KD-fed mice with AP. The overgrowth of *Mycoplasmatales* in feces and *Enterobacterales* in colonic tissue appears to explain the limitation of antibiotic treatment to aggravate acute pancreatitis. Butyrate supplementation attenuated the depletion of SCFAs, promoted the intestinal barrier, and reduced the necrotic area in AP mice. The bloom of *Bacteroidales* and the correlated increase in tryptophan metabolism explain the therapeutic potential of butyrate supplements for acute pancreatitis. In conclusion, our findings suggest that the ketogenic diet exacerbates acute pancreatitis through its impact on the gut microbiota and subsequent disruption of the intestinal barrier, while butyrate supplementation reverses this effect.

## 1. Introduction

The ketogenic diet (KD) is a dietary protocol characterized by extremely low carbohydrate consumption, resulting in increased ketone production. Originally developed as a therapeutic approach for epileptic patients unresponsive to anti-convulsant medication nearly a century ago [1,2], it has gained popularity in recent years as an expedited weight loss method [3]. However, the effects of a ketogenic diet on the host are still controversial. A study has indicated that the ketogenic diet leads to significant elevations in low-density lipoprotein and cholesterol levels [4], while others have revealed an augmented risk of colitis [5], inflammatory bowel disease (IBD) [6], and dysbiosis of the intestinal microbiota [7], highlighting its potential to induce inflammatory disorders within the digestive system. On the contrary, numerous studies confirmed that KD which induces a different distribution of macronutrients leads to positive effects in obesity [8], diabetes [9], and polycystic ovary syndrome (PCOS) [10].

Acute pancreatitis (AP) is a gastrointestinal disease characterized by necrosis in the acinar cells of the exocrine pancreas [11], precipitating inappropriate release and activation of trypsin and triggering the autodigestion of pancreatic parenchyma [12]. Notably, AP predominantly presented as a self-limiting disease. The secondary infection and storm of cytokines induced by the dysfunction of the intestinal barrier with its consequent translocation of gut bacteria were the pivotal aggravating factors in acute pancreatitis [12]. Meanwhile, the increased free fatty acids induced by diet is a high-risk factor in mediating end-organ failure [13,14] among several pathogenic mechanisms of acute pancreatitis. Our previous study demonstrated that KD induced disruption of the intestinal barrier, upregulation of cytokines in colonic tissue, and exacerbation of dextran sulfate sodium (DSS)-induced colitis [6]. These alterations induced by KD served as contributing factors in acute pancreatitis. Therefore, an estimation of the risks or benefits resulting from a ketogenic diet in acute pancreatitis is necessary. In the present study, the experimental acute pancreatitis was induced by intraperitoneal injection of L-arginine, which was widely used as a common pancreatitis model in rodents, causing irreversible damage to pancreatic mitochondria and the secondary necrosis vacuolization and inflammation [15,16]. Then, we attempted to rescue acute pancreatitis in this study through the administration of prophylactic antibiotics, which was a classic approach in clinical treatment for acute pancreatitis but controversial, or butyrate supplement which was rapidly decreased in mice fed with a KD. Amylase, lipase, and lipopolysaccharides-binding protein (LBP) in serum were measured to assess the severity of pancreatitis. Then, the intestinal barrier was estimated by the immunofluorescence assay and qPCR analysis. 16s rRNA amplicon sequencing, 2bRad-M sequencing, and microbial metabolomics were performed to demonstrate changes in gut and tissue microbiota and their metabolic function. Finally, cytokines and chemokines in serum were assessed to evaluate the systemic inflammation.

## 2. Materials and Methods

### 2.1. Mice and Models of Acute Pancreatitis (AP)

Six- to eight-week-old SPF C57BL/6 mice were procured from Shanghai Laboratory Animal Company (SLAC) and acclimated under standard conditions (22 ± 2 °C, 12 h light/dark cycle) for a duration of 2 weeks prior to the commencement of the study procedures. The animals were provided ad libitum access to water and either a standard chow diet (SD) or ketogenic diet (KD, TD.96355; Table S1). The acute pancreatitis model was induced by hourly intraperitoneal injection of L-arginine (4 g/kg body weight, pH = 7.3, MCE, HY-N0455), administered a total of two times. After 72 h following i.p. injection, the animals were euthanized under isoflurane anesthesia (RWD Life Science, Shenzhen, China). All animal experiments performed in this study were granted ethical approval by the Animal Ethics Care Committee of the First Affiliated Hospital, School of Medicine, Zhejiang University (permit number: 2023-1344).

### 2.2. Treatments

The germ-depletion (GD) option was implemented through oral gavage of a compound recipe consisting of four antibiotics. Specifically, 200 mg/kg ampicillin (MCE, HY-B0522, Dallas, TX, USA), 200mg/kg neomycin (MCE, HY-B0470, Dallas, TX, USA), 200 mg/kg metronidazole (Meilun biotech, MB2200-1, Dalian, China), and 100 mg/kg vancomycin (Meilun biotech, MB1260-3, Dalian, China) were administered. The control groups received regular drinking water.

Butyrate supplementation was conducted in two stages. For the oral supplement, butyric acid (100 mM, Sigma, B103500, St. Louis, MO, USA) was added to light-protected bottles containing drinking water for a duration of 4 weeks. A fresh solution of butyric acid was prepared every third day. Considering the dietary difficulties of the mice after L-arginine-induced pancreatitis, butyrate supplementation was performed by intraperitoneal injection, in which sodium butyrate (40 mM) was dissolved in PBS and administered per 24 h after modeling at the volume of 200 μL.

### 2.3. β-Hydroxybutyric Acid (BHB) Assessment

Blood samples were obtained from the mice’s tails by making a small incision using specialized scissors. The initial drop of blood was discarded, and the subsequent drop was analyzed using an Abbott taster (FreeStyle Optium Neo, Abbott, Hannover, Germany) equipped with compatible BHB test paper.

### 2.4. Assessment in Serum and Tissue

The levels of serum cytokines were measured using a 23-Plex Assay Kit (Bio-Plex Pro mouse cytokine 23-Plex panel; Bio-Rad, Hercules, CA, USA) and analyzed with the Magpix system (Luminex Corporation, Austin, TX, USA) and Bio-Plex Manager 6.1 software (Bio-Rad, Hercules, CA, USA), following the manufacturer’s instructions. The serum lipopolysaccharides-binding protein concentration was quantified using a mouse LBP ELISA Kit (ab269542, Abcam, Cambridge, UK). The serum amylase and lipase were detected by the Amylase Assay Kit (ab102523, Abcam, Cambridge, UK) and the Lipase Assay Kit (ab102524, Abcam, Cambridge, UK). Superoxide dismutase (SOD) concentration was quantified using an SOD Assay Kit (WST-1 method) (A001-3-2, Nanjing Jiancheng Bioengineering Institute, Nanjing, China), and the malondialdehyde (MDA) concentration was assessed by Trace Malondialdehyde (MDA) Test Kit (KGT004-1, KeyGEN biotech, Jiangsu, China).

### 2.5. Histology and Immunofluorescence Staining

The colon and pancreas samples were fixed in 4% paraformaldehyde (PFA) before analysis. Following paraffin embedding, the tissues were sectioned at 5 μm thickness for subsequent hematoxylin and eosin (H&E) staining. To assess the integrity of the intestinal barrier, colonic specimens were stained with Zo-1 (dilute 1:100, Ab221547, Abcam, Cambridge, UK) and occludin (dilute 1:100, Ab216327, Abcam, Cambridge, UK) antibody to evaluate the intestinal barrier. Fluorescence images were captured using the Zeiss LM710 confocal system with the software ZEN blue 3.1 (Zeiss, Jena, Germany).

### 2.6. Electron Microscopy (TEM)

Electron microscopy was performed on 80 nm sections of pancreatic tissue from mice. All the sections were fixed in 2.5% glutaraldehyde (Sigma, G5882, Gillingham, UK) in 0.1 M phosphate buffer. The pancreas tissues were dehydrated using incremental concentrations of ethanol solutions and pure acetone before being embedded in the Spurr embedding medium. Subsequently, ultrathin slices were obtained using an Ultramicrotome Leica EM UC7 (Leica, Wetzlar, Germany) and stained with lead citrate and uranyl acetate. The mucosal ultrastructure was observed using a Hitachi H-7650 transmission electron microscope (Hitachi–Science & Technology, Tokyo, Japan).

### 2.7. Real-Time qPCR Analysis

Total RNA was extracted from the colon tissues by the RNeasy plus Minikit (Qiagen, Hilden, Germany) and was immediately reverse-transcribed to cDNA and stored at −80 °C until further procedures. mRNA relative levels were measured in duplicate with TB Green Premix Ex Taq Reagent (TAKARA, Kusatsu, Japan) using an Applied Biosystems VIIA7 Real-time PCR system (Applied Biosystems, Waltham, MA, USA), and threshold cycle (^ΔΔCT^) was finally calculated to analyze the significant difference between groups. Primers are displayed in Table S2.

### 2.8. 16s rRNA Gene Sequencing

We extracted the feces genome DNA with a DNeasyPowerSoil Kit (QIAGEN, Hilden, Germany). The quality and concentration of the extracted DNA were assessed with a NanoDrop 2000 (Thermo Fisher Scientific, Waltham, MA, USA). The 16s rRNA gene sequencing was performed on the Illumina NovaSeq 6000 platform with paired-end reads of 250 bp length (Illumina Inc., San Diego, CA, USA; OE Biotech Company; Shanghai, China). The FLASH (V 1.2.7) software and QIIME2 were used to merge the paired-end reads and filter the high-quality clean sequences. After removing chimeric sequences, the effective tags were clustered into the amplicon sequence variants (ASVs) with Usearch10.0 (from www.drive5.com/usearch). Each representative sequence within each ASV was taxonomically annotated utilizing the GreenGene Database through an RDP classifier analysis tool (Version 2.2, San Diego, CA, USA). Finally, we conducted statistical analyses to assess microbial diversity and differential enrichment patterns.

### 2.9. 2bRad-M Gene Sequencing and Analysis

The 2bRAD-M library preparation closely followed the original protocol developed by Wang et al. (2bRad) [17], with minor modifications. Briefly, genomic DNA ranging from 1 pg to 200 ng was digested using 4U of BcgI enzyme (NEB) at 37 °C for a duration of 3 h. The resulting digested product was confirmed through agarose gel electrophoresis on a 1% gel. Subsequently, ligation reactions were carried out using library-specific adaptors (Ada1 and Ada2), followed by amplification in a 40 ul PCR for subsequent gene sequencing on the Illumina HiSeq platform. Purification of PCR products was performed using the QIAquick PCR Purification Kit (Qiagen, Hilden, Germany).

To identify microbial species within each sample, all sequenced 2bRAD tags after quality control were aligned against the 2bRAD marker database (based on microbial genomes from the NCBI RefSeq database v220 released in Sep 11, 2023), which encompasses all theoretically unique 2bRAD tags for each of the 26,163 microbial species. To mitigate false-positive identifications of species, a G score was calculated for each identified species within a sample as follows: It represents the harmonic mean of read coverage across 2bRAD markers specific to that species and the total number of possible 2bRAD markers associated with said species. A threshold G score value of 5 was established to minimize false-positive discoveries of microbial species [18].

Subsequently, we calculated the average read coverage across all 2bRAD markers for each individual species to estimate the number of individuals belonging to that species present in a given sample at a specific sequencing depth. The relative abundance of any given species was then determined by calculating the ratio between the number of individuals assigned to that specific microbial taxon and the total count of detectable individuals from known taxa within a given sample. Primers and adaptor sequences utilized are given in Table S2.

$${G\;score}_{species\;i}=\sqrt{S_{i}\times t_{i}}{Relative\;abundance}_{species\;i}=\frac{S_{i}\times T_{i}}{\sum_{i=1}^{n}S_{i}/T_{i}}$$

*S*: the number of reads assigned to all 2bRAD markers belonging to species *i* within a sample.
*t*: number of all 2bRAD markers of species *i* that have been sequenced within a sample.

### 2.10. Targeted (Short-Chain Fatty Acids) and Untargeted Metabolomics

Before sacrifice, fecal samples from mice were collected, snap-frozen, and stored at −80 °C. The measurement of short-chain fatty acids (SCFAs) was performed using a Trace 1300 gas chromatograph (Thermo Fisher, Waltham, MA, USA) equipped with an Agilent HP-INNOWAX capillary column (30 × 0.25 mm IDXO.25 μm). Metabolite detection by mass spectrometry was conducted on the ISQ 7000 platform (Thermo Fisher, Waltham, MA, USA). Untargeted GC-LC dual-platform mass spectrometry analysis was carried out using the Aglient-GCMS-7890B/5977A platform and Acquity UPLC I-Class plus system. 

### 2.11. Statistical Analysis

Statistical analysis was performed by GraphPad Prism 8 and R (version 4.0.3). All data were presented as means ± SD. The two-tailed unpaired Student’s *t*-test was utilized for comparing two groups. For the analyses, one-way ANOVA with Tukey’s post hoc test or Kruskal–Wallis test was conducted. A significance level of *p* < 0.05 was considered statistically significant.

## 3. Results

### 3.1. Ketogenic Diet Exacerbated L-Arginine-Induced Acute Pancreatitis and Induced Dysfunction of the Intestinal Barrier

**Figure 1:**
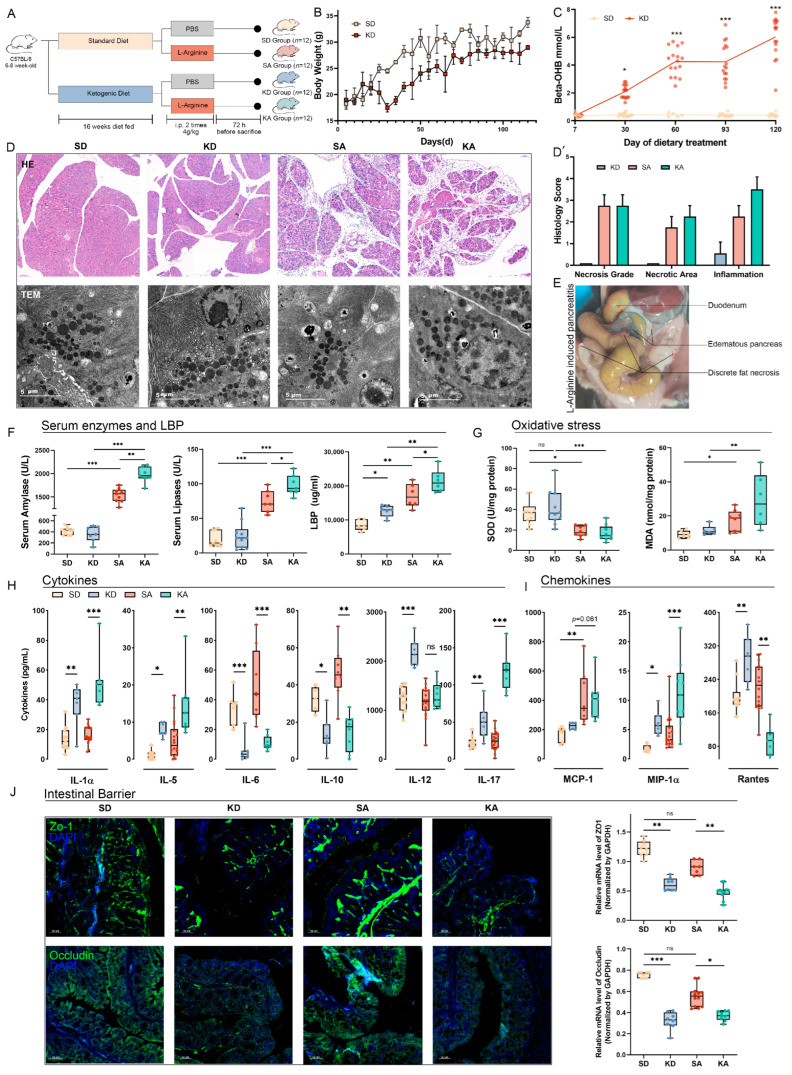
A ketogenic diet exacerbated L-arginine-induced acute pancreatitis and induced dysfunction of the intestinal barrier. (**A**) Scheme of L-arginine-induced pancreatitis in KD-fed mice. (**B**) Body weight for SD and KD-fed mice. (**C**) Beta-OHB in serum in SD and KD groups. (**D**) H&E staining and TEM sections of pancreatic tissue. (**D’**) Histology score of H&E staining assessed according to Schmidt’s criteria (*p >* 0.05 by Mann–Whitney test). (**E**) Macroscopic view of fatty necrosis. (**F**) Amylase, lipase, and LBP measurement. (**G**) SOD and MDA in pancreatic tissue to assess oxidative stress levels. (**H**) Cytokines in serum. (**I**) Chemokines in serum. (**J**) immunofluorescence sections and the expression levels of tight junction protein (Zo-1 and occludin both in green) in colonic tissue. All data were shown with mean ± SD, * *p* < 0.05, ** *p* < 0.01, *** *p* < 0.001.

To estimate the influence of the ketogenic diet on acute pancreatitis (AP), a long-period ketogenic diet (KD) was followed for 16 weeks, and the acute pancreatitis was induced by intraperitoneal injection of L-arginine (Arg) as the scheme (Figure 1A). For mice not induced with AP, the body weight was measured every 5 days, and the results demonstrated a significant downregulation of body weight in mice fed a KD within 1 month and remained lower than mice fed a standard diet (SD) consistently (Figure 1B). Additionally, the concentration of beta-OHB, a ketone body, gradually increased in venous blood and reached the threshold for ketonemia (>1 mmol/L) within 1 month compared to the SD group (*p* < 0.05) (Figure 1C). The H&E-stained sections revealed the presence of microscopic focal pancreatic necrosis and infiltration of inflammatory cells, while TEM sections highlighted ultrastructural dysfunction of mitochondria in exocrine pancreatic cells (Figure 1D). A histology score was calculated based on Schmidt’s criteria, which included grading necrosis, measuring necrotic area, and assessing inflammation (Figure 1D’). Discrete fat necrosis and swollen pancreatic tissue were observed in the SA and KA groups in a macroscopic view of the pancreas (Figure 1E). These findings demonstrate the induction of L-arginine-induced acute pancreatitis, characterized by widespread inflammation and necrosis of the pancreas.

The presence of amylase and lipase in serum serves as two well-established indicators for acute pancreatitis [16]. Our results demonstrated a significant increase in both enzymes following Arg-induced pancreatitis (*p* < 0.001), with the KA group exhibiting a higher elevation of amylase compared to the SA group. Additionally, an upregulation of lipopolysaccharide-binding protein (LBP) was observed in both the SA and KA groups (Figure 1F). Furthermore, the dysfunction of mitochondria suggests the presence of oxidative stress in pancreatic tissue. To validate this hypothesis, we assessed tissue SOD and MDA levels (Figure 1G). The results revealed a decrease in SOD activity and an increase in MDA concentration within the pancreatic tissue, indicating elevated production of oxygen free radicals and depletion of antioxidants.

To explore systemic inflammation in Arg-induced acute pancreatitis, cytokines and chemokines were assessed in our study (Figure 1H). Our results demonstrated an increase in pro-inflammatory factors such as IL-1α, IL-5, and IL-12 in the KD and KA groups compared to those fed an SD. Surprisingly, we observed a significant downregulation of IL-6, which is known to promote acute-phase proteins, in the KD and KA groups. This observation can be attributed to the extended period before sacrifice (72 h) rather than the routine 24 h duration. Meanwhile, anti-inflammatory factors like IL-10 and IL-17 were downregulated in mice fed a KD. For chemokines, MCP-1 and MIP-1α were increased both in the SA and KA groups, but Rantes was downregulated dramatically in the KA group (Figure 1I).

Gut microbiota is the primary source of secondary infection of pancreatic tissue [19], and the permeability of the intestinal barrier plays a central role. Our immunofluorescence (IF) staining and real-time qPCR results revealed a significant downregulation (*p* < 0.01) of Zo-1 and occludin, two proteins involved in maintaining tight junctions within the intestinal barrier (Figure 1J). Importantly, although there was a slight decrease in Zo-1 and occludin expression in the SA group compared to the SD group, this difference did not reach statistical significance (*p >* 0.05). The present findings suggest that the dysfunction of the intestinal barrier primarily arises from the ketogenic diet rather than L-arginine-induced pancreatitis. 

### 3.2. Ketogenic Diet Altered the Gut Microbiota in L-Arginine-Induced Acute Pancreatitis

**Figure 2:**
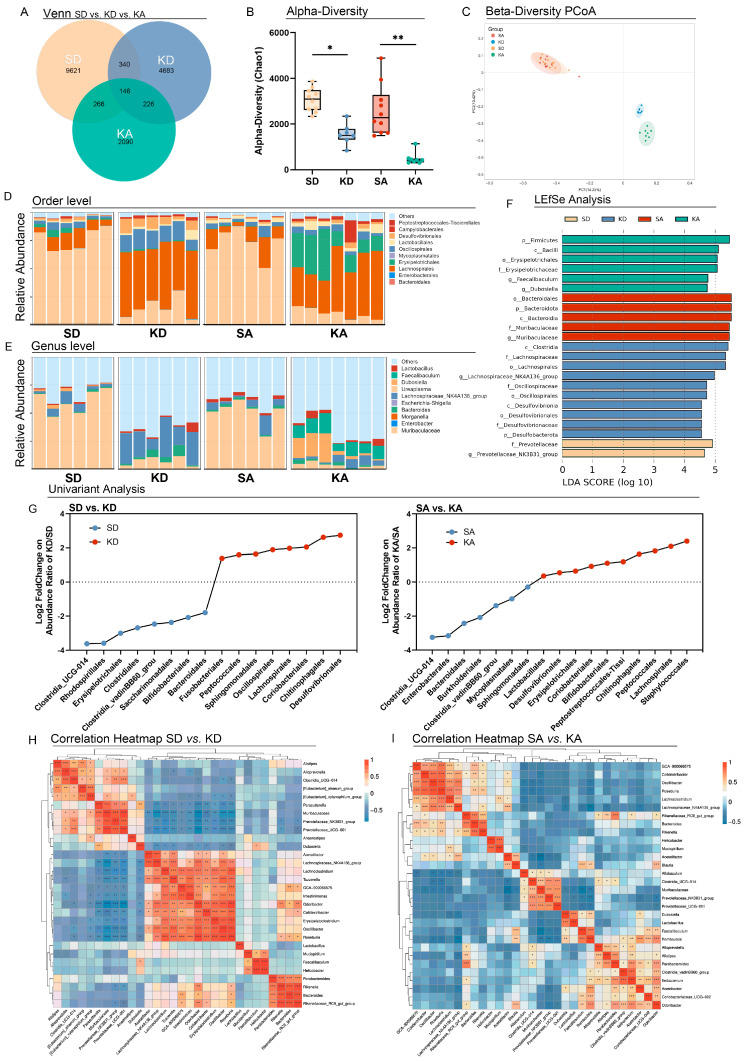
A ketogenic diet altered the gut microbiota in L-arginine-induced acute pancreatitis. (**A**) Venn analysis in SD, KD, and KA groups to show the overlap of gut microbiota. (**B**) Alpha diversity in SD, KD, SA, and KA groups. (**C**) Beta diversity in SD, KD, SA, and KA groups. (**D**) Relative abundance of gut microbiota at the order level. (**E**) Relative abundance of gut microbiota at the genus level. (**F**) LEfSe analysis shows the indicators among SD, KD, SA, and KA groups. (**G**) Univariant analysis to show the significantly different microbiota in SD vs. KD and SA vs. KA groups. (**H**) Correlation heatmap of SD vs. KD. (**I**) Correlation heatmap of SA vs. KA. All data were shown with mean ± SD, * *p* < 0.05, ** *p* < 0.01, *** *p* < 0.001.

Venn analysis among the SD, KD, and KA groups revealed a dynamic alteration of gut microbiota, with a relatively smaller proportion of shared ASVs observed across all three groups (Figure 2A). To validate this modification, we assessed alpha diversity in the gut microbiota. Our findings revealed a significant reduction in diversity among groups fed a ketogenic diet compared to those on standard and standard ad libitum diets (Figure 2B). Beta diversity analysis by the weighted UniFrac method suggested the distinct changes in gut microbiota between mice fed a KD and SD (Figure 2C). There were minimal differences observed in the abundance structure of gut microbiota between the SA and SD groups, while the difference in abundance structure between the KA and KD groups was also negligible. The 16s gene amplicon analysis demonstrated the relative abundance of feces microbiota in mice fed SD and KD (Figure 2D). At the order level, the main composition of microbiota in the SD and SA groups is *Bacteroidales*, whereas *Lachnospirales* and a part of *Erysipelotrichales* in KA and *Oscillospirales* in KD were bloomed. More specifically, at the genus level, *Muribaculaceae* is the main composition of *Bacteroidales* and *Lachnospiraceae NK4A136 group* representatives *Lachnospirales*. *Dubosiella* and *Faecalibaculum* are the two major compositions of *Erysipelotrichales* (Figure 2E).

An LEfSe analysis was conducted to identify the indicators of differential groups (Figure 2F). Corresponding to the relative abundance, *Firmicutes* at the phylum level and its members *Dubosiella* and *Faecalibaculum* are the specific indicators of the KA group, and *Bacteroidales* is the symbol of the SA group (log10 LDA score > 4.5). Univariant analysis according to the log2 fold change of microbiota was conducted to assess the low-abundant taxa with dramatic changes (Figure 2G). The results showed an increase in *Clostridiales*, *Saccharimonadales*, and *Bifidobacterales* in the SD group compared to the KD group, and an increase in *Desuifovibrionales*, commonly seen as a pathogenic bacterium in the KD group. Notably, the sphingomyelin-related taxa such as *Chitinophagales* and *Sphingomonadales* increased in the KD group, which appears to be a feature induced by the ketogenic diet. Moreover, *Mycoplasmatales* increased in the SA group. In summary, the ketogenic diet induced a shift of gut microbiota from *Bacteroidales* to *Lachnospirales* and *Erysipelotrichales*, and a part of low-abundance pathogens increased in KD-fed mice. The correlated analysis of gut microbiota reveals five main clusters in the SD and KD groups (Figure 2H). *Lachnospirales*, *Oscillibacter*, and *Erysipelotrichales* form a large synergistic cluster and show a strong negative correlation with another cluster composed of *Muribaculaceae*, *Parasutterella*, and *Prevotellaceae*. This antagonistic effect continues into the comparison between SA and KA. Additionally, the correlation between *Firmicutes* and *Lachnospiraceae NK4A136 group* was reversed from positive in SD and KD comparison to negative between SA and KA (Figure 2I). These results suggest that the main conflict in KD-fed mice is between a cluster composed of *Lachnospirales* and *Firmicutes*, and another cluster composed of *Prevotellaceae*, whereas L-arginine-induced pancreatitis reverses the synergistic relationship between *Firmicutes* and *Lachnospirales*.

### 3.3. Ketogenic Diet Modulated Gut Metabolism and Resulted in the Depletion of Short-Chain Fatty Acids

Metabolism plays a central role in the interaction between gut microbiota and host [20]. Hence, metabolomics analysis was performed to assess the alteration in metabolites induced by the ketogenic diet or L-arginine stimulation.

**Figure 3:**
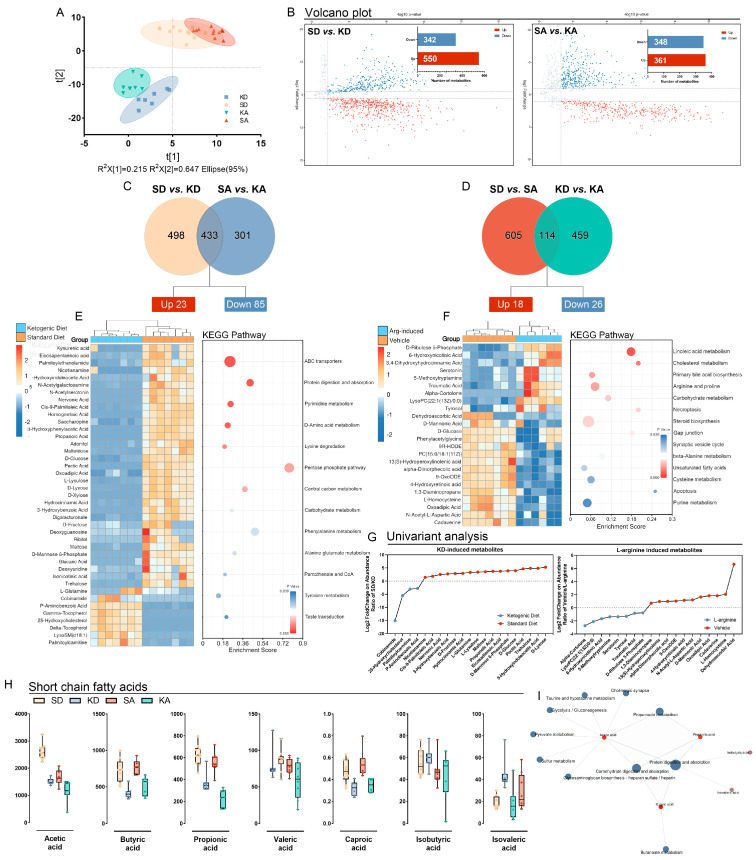
A ketogenic diet modulated gut metabolism and resulted in the depletion of short-chain fatty acids. (**A**) PLS-DA among KD, SD, SA, and KA groups. (**B**) Volcano plot of metabolites in SD vs. KD and SA vs. KA. (**C**) Venn analysis between SD vs. KD and SA vs. KA to show the overlapped metabolites resulting from KD. (**D**) Venn analysis between SD vs. SA and KD vs. KA to show the overlapped metabolites resulting from L-arginine injection. (**E**) Altered metabolites associated with ketogenic diet. (**F**) Altered metabolites associated with L-arginine injection. (**G**) Univariant analysis for KD and L-arginine-induced metabolites. (**H**) Short-chain fatty acids assessment. (**I**) Network map established based on KEGG pathway enrichment. All data were shown with mean ± SD.

The differential metabolites among groups were detected by partial least squares discrimination analysis (PLS-DA). The results were consistent with the 16s rRNA-based compositional data, demonstrating a clear distinction between SD-fed and KD-fed mice (Figure 3A). The significantly different metabolites were defined with the criteria of *p*-value *<* 0.05 and log2 fold change > 2. In detail, compared with the SD group, 550 metabolites were upregulated and 342 metabolites were downregulated in the KD group, while there were 361 metabolites increased and 348 metabolites decreased in the KA group compared with the SA group (Figure 3B). Furthermore, there were 433 metabolites from the set of SD vs. KD intersected with the metabolites set of SA vs. KA (Figure 3C). This intersection represents the metabolite changes induced by KD after the exclusion of the effects caused by arginine-induced acute pancreatitis, in which 23 metabolites were increased and 85 were decreased.

According to the *p*-value, fold change, and VIP, 41 metabolites were listed in a heatmap. Compared with the SD group, carbohydrates (D-fructose, D-glucose, maltose, D-xylose, and mannose 6-phosphate), carbohydrate derivatives (digalacturonate, pectic acid, and glucaric acid), and unsaturated fatty acids (eicosapentaenoic acid and nervonic acid) were significantly downregulated in the KD group. Additionally, propanoic acid, known as one of the short-chain fatty acids (SCFAs), was decreased in KD-fed mice. Furthermore, functions of overlapped metabolite sets were explored by aggregating them into functional categories using the Kyoto Encyclopedia of Genes and Genomes (KEGG). The differential metabolites were predominantly enriched in pathways involved in ABC transporters, pentose phosphate pathway, protein digestion, and carbohydrate metabolism (Figure 3E). These results indicated that the influence of the ketogenic diet predominately relies on altering the ability of the microbial community to use carbon sources.

The intersection between SD vs. SA and KD vs. KA metabolites set was filtered (Figure 3D), and then 114 metabolites were observed, of which 18 were upregulated and 26 metabolites were downregulated. After the exclusion of insignificant items, 24 metabolites were displayed in a heatmap (Figure 3F). Carbohydrates and their derivatives (maltotriose, D-fructose, N-acetyl galactosamine, and D-glucose) were also depleted in the control group. Additionally, tryptophan and its derivatives such as serotonin and 5-methoxytryptamine were accumulated in mice with pancreatitis induced by L-arginine. KEGG pathway enrichment results demonstrated the significant changes in lipids-associated metabolism (linoleic acid, cholesterol, steroid, and unsaturated fatty acids) and amino acid metabolism (arginine, beta-alanine, and cysteine), which appears to be explained by inflammation and injection resulting from L-arginine. Notably, the enrichment of necroptosis and apoptosis pathways highlights the vital role of cell-death-related signaling pathways in L-arginine-induced acute pancreatitis.

To verify the reduction in propionic acid mentioned above, a targeted metabolomic analysis for SCFAs was performed (Figure 3H). The results demonstrated that acetic acid, butyric acid, propionic acid, caproic acid, and isovaleric acid were significantly decreased in the KD-fed group, but valeric acid and isobutyric acid were exceptions. SCFA-associated metabolic pathways were exhibited in the network map (Figure 3I).

### 3.4. Germ-Depletion and Butyrate Supplementation Treatment Attenuate Necrosis of Pancreatic Tissue and Systemic Inflammation in L-Arginine-Induced Acute Pancreatitis

**Figure 4:**
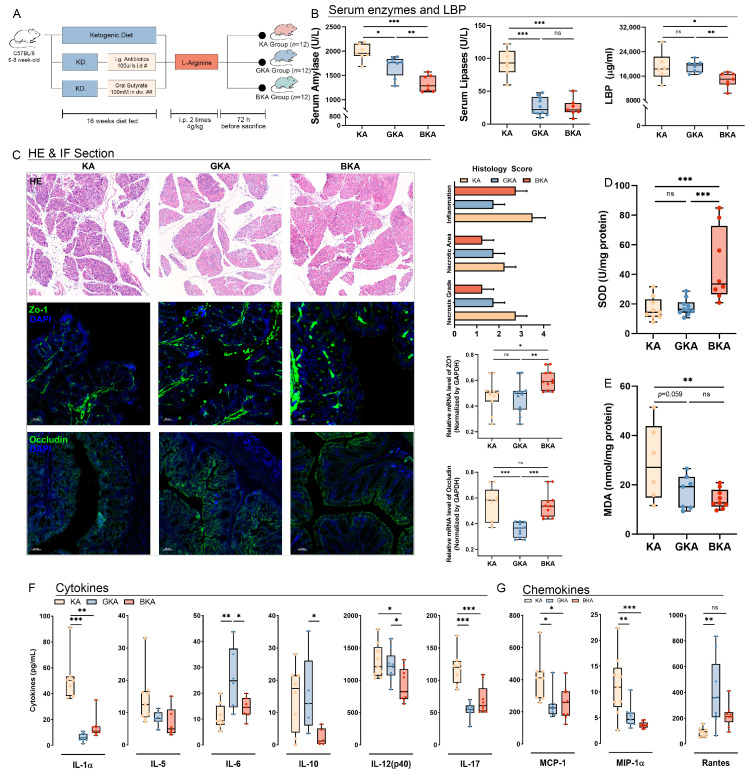
Germ-depletion and butyrate supplementation treatment attenuate necrosis of pancreatic tissue and systemic inflammation in L-arginine-induced acute pancreatitis. (**A**) Scheme of germ-depletion and butyric acid supplement groups. (**B**) Serum amylase, lipase, and LBP levels. (**C**) H&E-stained sections of the pancreas and IF sections of the intestinal barrier with expression levels of Zo-1 (green) and occludin (green) measured with qPCR. (**D**) SOD in pancreatic tissue. (**E**) MDA in pancreatic tissue. (**F**) Cytokines in serum. (**G**) Chemokines in serum. All data were shown with mean ± SD, * *p* < 0.05, ** *p* < 0.01, *** *p* < 0.001, ns = not significant.

Compared to the SD group, KD-fed mice exhibited spontaneous disruption of the intestinal barrier and elevated levels of serum cytokines, even in the absence of L-arginine-induced acute pancreatitis (Figure 1). In comparison to the SA group, abnormal increases were observed in serum amylase and pro-inflammatory factors (IL-1, IL-5, and MIP-1) in the KA group, with a lack of reactive increase in anti-inflammatory factors amidst systemic inflammation. Considering that KD significantly altered gut microbiota (Figure 2) and metabolic function (Figure 3), we hypothesized that gut microbiota might be implicated in exacerbating acute pancreatitis and inducing gut barrier dysfunction. To validate this hypothesis, a 7-day course of oral quadruple antibiotics (neomycin, ampicillin, metronidazole, and vancomycin) was administered to induce a germ-depletion (GD) for the KA group (GKA). SCFAs play a vital role as essential carbon sources for intestinal epithelial cells in an anaerobic environment [21], whereas the metabolic analysis of the KD and KA groups revealed a significant depletion of SCFAs. To investigate the impact of SCFAs’ depletion or repletion on the KA group, mice were administered 100 mM butyrate via drinking water for a duration of 30 days (BKA). The experimental procedures are illustrated in Figure 4A.

Serum amylase and lipase levels were measured to assess pancreatic tissue injury, and the results demonstrated a decrease in these markers following either germ-depletion or butyric acid supplementation (Figure 4B), and the serum amylase level was lower in the BKA group compared to the GKA group. The decreased LBP level in the BKA group (*p* < 0.05) indicated reduced endotoxin release induced by butyric acid supplementation, whereas it was not decreased in the GKA group (Figure 4B). To investigate pancreatic necrosis and intestinal barrier injury, we performed H&E and IF staining of pancreatic tissue, histology score assessment, as well as real-time qPCR analysis of Zo-1 and occludin expression levels (Figure 4C). The results revealed attenuation of pancreatic necrosis in both GKA and BKA groups. However, inflammatory infiltration attenuation was less efficient in the BKA group. Importantly, enhanced expression of Zo-1 and occludin in the BKA group indicated improved intestinal barrier function due to butyric acid supplementation. Conversely, germ-depletion resulted in downregulated expression of occludin (*p* < 0.001) without promoting Zo-1 expression compared with the KA group. Moreover, the butyric acid intervention significantly promoted SOD activity (*p* < 0.001) while decreasing MDA concentration (*p* < 0.01) within pancreatic tissue; however, these changes were not significant in the GKA group (Figure 4D,E).

Subsequently, the levels of cytokines and chemokines in serum were quantified to assess systemic inflammation following treatment with antibiotics and butyric acid. The results indicated a decrease in pro-inflammatory cytokines (IL-1a, IL-5, and IL-12) within both the GKA and BKA groups (Figure 4F). Interestingly, IL-6 showed an increase in the GKA group, suggesting that antibiotic-induced germ-depletion may not completely attenuate secondary infections as expected. Additionally, anti-inflammatory cytokines (IL-10 and IL-17) exhibited reduced expression specifically within the BKA group (Figure 4F). Furthermore, significant downregulation of chemokines such as MCP-1 and MIP-1a was observed in both the GKA and BKA groups, but Rantes displayed increased expression solely within the GKA group (Figure 4G).

### 3.5. Germ-Depletion and Butyrate Supplement Treatment Altered Gut and Colonic Microbiota

**Figure 5:**
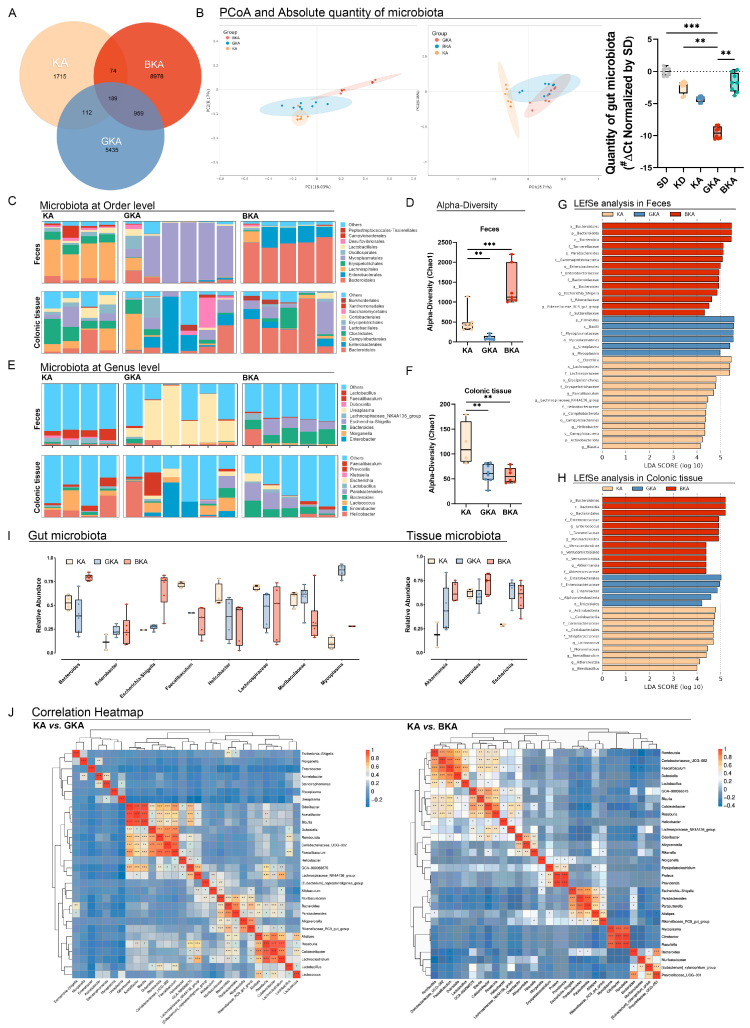
Germ−depletion and butyrate supplement treatment altered gut and colonic microbiota. (**A**) Venn analysis among KA, GKA, and BKA groups. (**B**) PCoA of microbiota in gut or colonic tissues, and the absolute quantity of gut microbiota. (**C**) Relative abundance of microbiota in feces and colonic tissue at the order level. (**D**) Alpha diversity in feces microbiome. (**E**) Relative abundance of microbiota in colonic tissue at the genus level. (**F**) Alpha diversity in colonic tissue microbiome. (**G**) LEfSe analysis of microbiota in feces. (**H**) LEfSe analysis of microbiota in colonic tissue. (I) Relative abundance of gut and tissue microbiota. (**J**) Correlation heatmap in GKA vs. KA and BKA vs. KA. # ΔCt in Figure 5B was calculated by “target Ct-mean Ct of SD group”. All data were shown with mean ± SD, * *p* < 0.05, ** *p* < 0.01, *** *p* < 0.001.

To assess the effect of antibiotics on reducing the abundance of gut microbiota and explore the reasons for inefficient influence on oxidative stress and intestinal barrier in the GKA group, sequencing based on 16s rRNA and 2bRad-M was performed in both gut and colonic tissues to demonstrate the relative abundance and diversity of the local microbiota. The Venn analysis demonstrated the impact of germ-depletion and butyric acid on gut microbiota using 16s rRNA sequencing (Figure 5A). This analysis filtered the common 189 ASVs among these three groups, and there were 74, 122, and 959 ASVs regulated in comparison of KA vs. BKA, KA vs. GKA, and BKA vs. GKA, indicating that the gut microbiota of GKA was more similar to the BKA group. Principal co-ordinates analysis (PCoA) and the absolute quantification of gut microbiota demonstrated significant differences in gut and colonic microbiota among KA, GKA, and BKA groups (Figure 5B) in both absolute and relative abundance. The quantity of gut microbiota reduced rapidly in the GKA group compared to all other groups in our study, and the quantity of microbiota increased after the supplementation of butyrate compared with the GKA group (Figure 5B). Moreover, the body weight of mice in the BKA group exhibited no significant alterations when compared to that of mice in the KA group (Figure S1). Relative abundance analysis of gut microbiota at the order level demonstrated the bloom of *Mycoplasmatales* in the GKA group, and the abundance of *Bacteroidales* was increased in the BKA group compared with the KA group (Figure 5C). Meanwhile, the bloom of *Enterobacterales* in the BKA group was observed in gut microbiota.

To assess the infection of colonic tissue and reflect the function of the intestinal barrier, 2bRad-M sequencing was performed. Different from the abundance of gut microbiota, *Enterobacterales* and *Saccharomycetales* were significantly increased in the colonic tissue of the GKA group (Figure 5C). On the contrary, the structure of abundance in colonic tissue of the BKA group was consistent with its gut microbiota, where *Bacteroidales* was dominated and *Enterobacterales* bloomed in feces did not prevail in colonic tissue, indicating the promotion of intestinal barrier resulting from the butyric acid supplementation. Furthermore, relative abundance analysis at the genus level revealed that specific taxa changed both in feces and colonic tissue. The bloom of *Mycoplasmatales* mainly consisted of *Ureaplasma* in the GKA group and *Escherichia-Shigella*, a pathogenic bacterium, was the main composition of *Enterobacterales* in the BKA group. Notably, *Escherichia* did not prevail in the colonic microbiota of the BKA group. Additionally, as demonstrated by the Chao1 index, alpha diversity was significantly decreased in the GKA group compared with SD, KD, and KA groups in both the feces and colonic tissue-related microbiota, indicating the specific impact of quadruple antibiotics on germ-depletion (Figure 5E,F). LEfSe analysis revealed the specific indicators of KA, GKA, and BKA groups both in the gut and colonic microbiota (Figure 5G,H). Consistent with the relative abundance analysis, the indicators of the KA group in gut microbiota were *Lachnospiraceae* and *Erysipelotrichaceae* families. *Mycoplasmataceae* dominated in the GKA group. The indicators of the BKA group were *Bacteroidales* and *Enterobacterales*.

For the gut microbiota, antibiotics treatment reduced the abundance of *Bacteroides* and dramatically increased the abundance of *mycoplasma*, demonstrating that simply depletion of bacteria led to a reduction in the selection pressure of gut microbiome and the propagation of low-abundance pathogenic bacteria (Figure 5I). Interestingly, the bloom of a famous pathogenic bacteria *Escherichia-Shigella* was observed in the BKA group, but it is not significant in tissue microbiota. Considering the promotion of alpha diversity in the gut microbiota of BKA, we inferred that butyric acid supplementation attenuated the systemic inflammation resulting from acute pancreatitis by reducing the migration of gut microbiota.

### 3.6. Germ-Depletion and Butyric Acid Treatment Remodeling the Gut Metabolism

**Figure 6:**
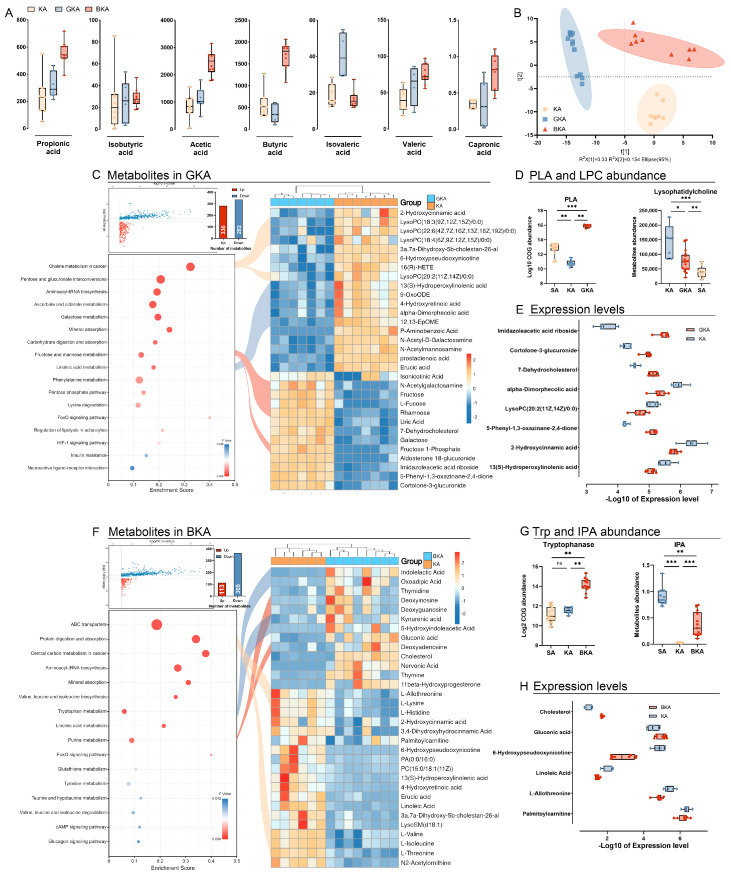
Germ−depletion and butyric acid treatment remodeling the gut metabolism. (**A**) SCFAs measurement. (**B**) PLS-DA among KA, GKA, and BKA groups. (**C**) Metabolites altered significantly between the KA and GKA groups. (**D**) LPC concentration and PLA abundance. (**E**) Expression level of metabolites between the KA and GKA groups. (**F**) Metabolites altered significantly between the KA and BKA groups. (**G**) Abundance of tryptophanase and concentration of IPA. (**H**) Expression level of metabolites between the KA and BKA groups. All data were shown with mean ± SD, * *p* < 0.05, ** *p* < 0.01, *** *p* < 0.001.

To investigate the potential interaction between gut microbiota and host tissue, we performed targeted metabolomics analysis of SCFAs and untargeted metabolomics analysis using GC-MS and LC-MS techniques. Firstly, our results demonstrated a significant increase in SCFA levels in mice treated with butyric acid supplement in the BKA group compared to the KA group, except for isobutyric acid and isovaleric acid (Figure 6A). Furthermore, PLS-DA analysis revealed distinct differences in metabolite profiles among the KA, GKA, and BKA groups (Figure 6B).

In detail, the GKA group exhibited a decrease in 282 metabolites and an increase in 336 metabolites compared to the KA group. Subsequently, 106 KEGG-annotated metabolites were selected for enrichment analysis based on VIP, FDR *p*-value, and fold change criteria. Among these metabolites, 18 downregulated and 13 upregulated ones were clustered into a heatmap (Figure 6C). Compared with the KA group, choline metabolism and linoleic acid metabolism were upregulated significantly in the GKA group, indicating the effects resulting from germ-depletion. The metabolites in both enhanced pathways were further analyzed, and the results demonstrated a significant increase in lysophosphatidyl choline (LysoPC) levels in the GKA group compared to the KA group (Figure 6C). Moreover, PICRUSt2 analysis of gut microbiota revealed a significant enrichment of phospholipase-A (PLA), known as an LPS producer, in the GKA group compared to the SA and KA groups (Figure 6C). Additionally, linoleic acid derivatives such as 13(S)-hydroperoxylinoleic acid (13(S)-HPODE), 9-OxoODE, and alpha-dimorphecolic acid were significantly reduced in the GKA group. This result was accompanied by an increased concentration of fructose, rhamnose, and fructose 1-phosphate, indicating a shift in carbon source utilization by gut microbiota.

Moreover, there were 365 decreased and 113 increased metabolites detected in the BKA group compared with the KA group. Based on the same criteria as the GKA group, 35 metabolites were filtered and included in the heatmap. Compared with the KA group, the essential amino acids (lysine, threonine, valine, and isoleucine) were significantly decreased in the BKA group and enriched in the protein digestion and absorption pathway. On the contrary, purine metabolism (deoxyinosine, deoxyadenosine, and deoxyguanosine) and tryptophan metabolism (kynurenic acid, oxoadipic acid, 5-hydroxyindoleacetic acid, 3,4-dihydroxyphenylglycol, and homovanillic acid) in the BKA group were upregulated significantly. PICRUSt2 analysis demonstrated the rapidly increased abundance of tryptophanase along with the increased concentration of indole-3-propionic acid (IPA), suggesting the accelerated metabolism of tryptophan and the synthesis of indole and its derivatives.

## 4. Discussion

The ketogenic diet refers to a dietary regimen with high-fat, low-carbohydrate, and adequate protein. The classic ketogenic diet delivers 90% of its calories from fat, 8% from protein, and only 2% from carbohydrates [22]. Our results are consistent with other studies, demonstrating the progressive increased serum beta-OHB levels and a sustained reduction in body weight compared to SD-fed mice for 16 weeks.

Obesity is commonly associated with impaired intestinal barrier function [23], and the impact of ketogenic diets on the intestinal barrier has been controversial in studies [5,6,24]. Therefore, it is imperative to thoroughly evaluate the potential risks of weight loss when implementing a ketogenic diet in patients.

Moreover, studies have consistently indicated that dietary constituents profoundly influence the abundance and diversity of gut microbiota [25]. Augmented intake of polysaccharides [26] has proven beneficial for sustaining the abundance of probiotics and the production of SCFAs, thereby playing a pivotal role in maintaining intestinal barrier integrity [27]. Our results indicate that following a ketogenic diet leads to a reduced abundance of Bacteroidetes, downregulation of pentose phosphate metabolism and carbohydrate metabolism pathways, and the depletion of SCFAs. Based on previous evidence highlighting the role of SCFAs, we propose that this reduction appears to serve as the primary potential factor contributing to decreased expression of tight junction proteins such as Zo-1 and Occludin.

Acute pancreatitis is a gastrointestinal disease characterized by systemic inflammation and necrosis of the exocrine tissue of the pancreas [28]. The majority of cases of acute pancreatitis were self-limiting, while 20% present severe [29]. Secondary infection of pancreatic tissue resulting from the dysfunction of the intestinal barrier is a fatal complication of acute pancreatitis [30]. Therefore, we established an L-arginine-induced acute pancreatitis model with an extended post-induction survival time (72 h) to assess the potential risks in KD-fed mice. Our results revealed that KD exacerbated inflammatory infiltration, necrosis of pancreatic exocrine (evidenced by increased serum amylase and lipase levels), and increased cytokines in serum. Interestingly, although serum LBP level did not significantly increase in KD-fed mice, it exhibited a more pronounced elevation compared with the SD group in the acute pancreatitis model. Previous studies on obesity models have suggested a correlation between alterations in permeability, glucose metabolism, and serum endotoxin levels [31].

To investigate the role of gut microbiota in the interaction between KD-fed and acute pancreatitis, germ-depletion in gut luminal microbiota was performed by quadruple antibiotics. The benefit of prophylactic antibiotics in reducing the morbidity of secondary infection and necrosis in pancreatic tissue is controversial in clinical trials. Some meta-analyses showed that prophylactic antibiotics were significantly associated with reduced all-cause mortality in patients with severe pancreatitis [32,33], while other studies negated the correlation between mortality and prophylactic antibiotic treatment [34,35,36]. In the present study, bacterial clearance drastically reduced alpha diversity and absolute quantification of intestinal microbiota in mice, reduced pancreatic tissue necrosis and cytokines levels, but did not improve the intestinal barrier.

The competition for host-derived nutrients and bacterial-derived metabolites in different gut microbiota constituted the habitat filters, which ultimately inhibit the migration and proliferation of low-abundance pathogens [37]. In the present study, the use of antibiotics inevitably disrupted the selective pressure of gut microbiota, leading to the bloom of *Enterobacteriaceae* and *Mycoplasma*, but downregulating lysophosphatidylcholine (LysoPC) and its derivation. Interestingly, a study has confirmed that the increased LysoPC aggravates experimental colitis [38], which appears to be the mechanism of prophylactic antibiotics to attenuate acute pancreatitis. In summary, our results supported that antibiotic decontamination is beneficial for L-arginine-induced acute pancreatitis, but not totally efficient in preventing secondary infection resulting from the translocation of gut microbiota, which needs more investigation.

In contrast to antibiotic decontamination, supplementation of butyrate in KD-fed mice with acute pancreatitis resulted in enhanced integrity of the intestinal barrier, significantly reduced pancreatic tissue necrosis and serum LBP levels, and attenuated oxidative stress in pancreatic tissue. SCFAs, mainly synthesized by gut microbiota in the anaerobic environment [39], maintained the integrity of the gut barrier by regulating mucus production and providing fuel for epithelial cells [40] and had been shown to promote tight junction protein expression [41,42]. Additionally, the enrichment of the tryptophan metabolism pathway and increased indole production revealed the upregulation of microbial pathways involved in tryptophan metabolism within the BKA group. A previous study demonstrated an inverse correlation between tryptophan levels and the activity of inflammatory bowel disease [43]. Tryptophan metabolites such as IPA, indoleacetic acid (IA), and indole-ethanol (IE) attenuated the dysfunction of the intestinal barrier by activating toll-like receptor 4 (TLR-4) and xenobiotic sensor pregnane X receptor (PXR) [44], which increased the secretion of products from goblet cells. The observed attenuation of the intestinal barrier in the BKA group appears to be explained by this mechanism, but further investigation is necessary.

Previous studies have demonstrated that induced modulation of gut microbiota has been associated with improvements in various experimental disease models [45]. There are three levels of methods to alter the gut microbiota, including the provision of prebiotics [46] or antibiotics [47] to regulate the target gut microbiota, the direct transfer through fecal microbiota transplantation (FMT) [48] or probiotic preparations [49], and the treatment of bacterial metabolites or extracellular macromolecules, called postbiotic, to simulate the interaction between the microbiota and the host [50]. However, the effects of FMT or multiple probiotic administration on improving intestinal barrier function, maintaining intestinal homeostasis, and ameliorating systemic inflammation are heterogeneous in clinical trials [51,52]. Our study also confirmed that antibiotic decontamination may increase the risk of pathogen infection. Therefore, we propose utilizing multi-omics combined analysis and in vivo validation to elucidate the mechanisms underlying microbiota–host interactions and suggest that corresponding postbiotic preparation represents a promising research direction for future studies.

There are still several limitations in this study. Firstly, although the utilization of antibiotic decontamination experiments has established the association between gut microbiota and the exacerbation of acute pancreatitis caused by a ketogenic diet, further investigation is required to elucidate the underlying mechanisms involved. Secondly, our findings do not provide evidence regarding whether discontinuation of the ketogenic diet can ameliorate acute pancreatitis exacerbation. This aspect needs to be explored to make informed decisions about the clinical implementation of ketogenic diets.

## 5. Conclusions

In conclusion, our study demonstrates that KD contributes to intestinal barrier dysfunction by modulating the composition of gut microbiota, leading to a depletion of SCFAs. This result appears to explain the elevated levels of serum amylase, lipase, and cytokines in mice fed with KD in L-arginine-induced acute pancreatitis. The administration of prophylactic antibiotics ameliorated pancreatic tissue necrosis and systemic inflammation but did not mitigate the dysfunction of the intestinal barrier and led to the overgrowth of *mycoplasma*. Meanwhile, supplementation with butyrate effectively mitigated systemic inflammation, enhanced the integrity of the intestinal barrier, and ultimately ameliorated L-arginine-induced acute pancreatitis.

## Data Availability

The data of 16s rRNA sequencing are available in ID 1028574—BioProject—NCBI (nih.gov). The data presented in the study are available in the article.

## Supplementary Materials

The following supporting information can be downloaded at https://www.mdpi.com/article/10.3390/nu15204427/s1, Table S1: Details of ketogenic diet. Table S2: Adaptors and primers used for Real-time qPCR and 2bRad-M preparation. Figure S1: Body weight of KA and BKA. Alteration of body weight for KA and BKA group to shown the influence of butyrate supplement.
