# Supplementary material for: Ketogenic Diet Exacerbates L-Arginine-Induced Acute Pancreatitis and Reveals the Therapeutic Potential of Butyrate
Source: Nutrients. 2023 Oct 18;15(20):4427. doi: 10.3390/nu15204427 (PMC10609823; doi:10.3390/nu15204427)
Supplement: Supplementary file 1 [file nutrients-15-04427-s001.zip › nutrients-2638538-supplementary.pdf]

Supplement Tables:

Table S1. Details of ketogenic diet

**Formula**	**g/Kg**
**Source of protein**	
Casein	173.3
DL-Methionine	2.6
**Source of fat**	
Vegetable Shortening, hydrogenated	586.4
Corn Oil	86.2
**Additives**	
Cellulose	87.97
Vitamin Mix, Teklad (40060)	13.0
Choline Bitartrate	2.5
TBHQ, antioxidant	0.13
Mineral Mix, Ca-P Deficient (79055)	20.0
Calcium Phosphate, dibasic	19.3
Calcium Carbonate	8.2
Magnesium Oxide	0.4

**Nutrient infomation**		
	% by weight	% kcal from
**Protein**	15.3	9.2
**Carbohydrate**	0.5	0.3
**Fat**	67.4	90.5

Table S2. Adaptors and primers used for Real-time qPCR and 2bRad-M preparation

**Primers in qPCR**		
**Gene**	**Forward sequence (5'-3')**	**Reserve sequence (3'-5')**
GAPDH	CCCTTAAGAGGGATGCTGCC	ACTGTGCCGTTGAATTTGCC
Zo1	GCCGCTAAGAGCACAGCAA	GCCCTCCTTTTAACACATCAGA
Occludin	TGAAAGTCCACCTCCTTACAG	CCGGATAAAAAGAGTACGCTG
Quantity primers	GTGCCAGCMGCCGCGG	TTTGARTTTMCTTAACTGCC
**Primers and adaptors in 2bRad-M**		
**Primers**	**Sequence (5'-3')**	
Primer1	ACACTCTTTCCCTACACGACGCT	
Primer2	GTGACTGGAGTTCAGACGTGTGCT	

Primer3	AATGATACGGCGACCACCGAGATCTACACTCTTTCCCTACACGACGCT	
Index primer	CAAGCAGAAGACGGCATACGAGATXXXXXXGTGACTGGAGTTCAGACGTGT	
**Adaptors in 2bRad-M**		
**Adaptors**	**Sense (5'-3')**	**Antisense (5'-3')**
Adap1	ACACTCTTTCCCTACACGACGCTCTTCCGATCTNNN	AGATCGGAAGAGC(AminoC6)
Adap2	GTGACTGGAGTTCAGACGTGTGCTCTTCCGATCTNNN	AGATCGGAAGAGC(AminoC6)

Supplement Figure:

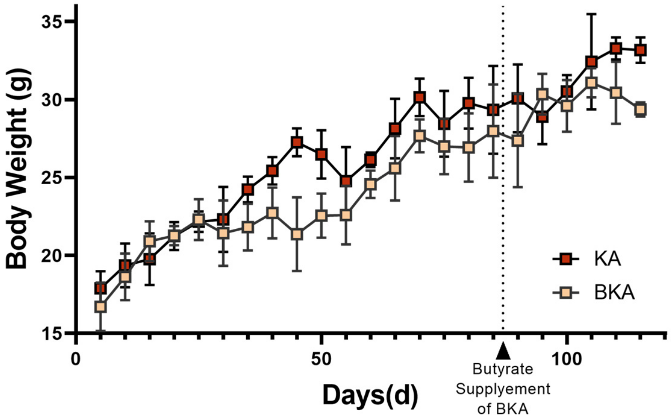

Figure S1. Body weight of KA and BKA

Alteration of body weight for KA and BKA group to shown the influence of butyrate supplement.
